# Safety and effectiveness of coronary intravascular lithotripsy in eccentric calcified coronary lesions: a patient-level pooled analysis from the Disrupt CAD I and CAD II Studies

**DOI:** 10.1007/s00392-020-01737-3

**Published:** 2020-09-18

**Authors:** Florian Blachutzik, Benjamin Honton, Javier Escaned, Jonathan M. Hill, Nikos Werner, Adrian P. Banning, Alexandra J. Lansky, Sophia Schlattner, Bernard De Bruyne, Carlo Di Mario, Oliver Dörr, Christian Hamm, Holger M. Nef

**Affiliations:** 1 Department of Cardiology, Medical Clinic I, University Hospital Giessen, Justus Liebig University Giessen, Klinikstrasse 33, 35392 Giessen, Germany; 2 Clinique Pasteur, Toulouse, France; 3 Hospital Clínico San Carlos IDISSC, Complutense University of Madrid, Madrid, Spain; 4 King’s College Hospital, London, UK; 5 Krankenhaus der Barmherzigen Brüder Trier, Trier, Germany; 6 Department of Cardiology, Oxford University Hospitals, Oxford, UK; 7 Yale University Medical Center, New Haven, USA; 8 Department of Cardiology, Cardiovascular Research Centre, OLV Hospital, Aalst, Belgium; 9 Structural Interventional Cardiology, Careggi University Hospital, Florence, Italy

**Keywords:** Lithotripsy, Clinical research, Calcified lesions, Percutaneous coronary intervention

## Abstract

**Background:**

The aim of this study was to assess the safety and effectiveness of intravascular lithotripsy (IVL) in treating eccentric calcified coronary lesions.

**Methods:**

Between December 2015 and March 2019, 180 patients were enrolled in the Disrupt CAD I and CAD II studies across 19 sites in 10 countries. Patient-level data were pooled from these two studies (*n* = 180), within which 47 eccentric lesions (26%) and 133 concentric lesions were identified.

**Results:**

Clinical success, defined as residual stenosis < 50% after stenting and no in-hospital MACE, was similar between the eccentric and concentric cohorts (93.6% vs. 93.2%, *p* = 1.0). There were no perforations, abrupt closure, slow flow or no reflow events observed in either group, and there were low rates of flow-limiting dissections (Grade D–F: 0% eccentric, 1.7% concentric; *p* = 0.54). Final acute gain and percent residual stenosis were similar between the two groups. Final residual stenosis of 8.6 ± 9.8% in eccentric and 10.0 ± 9.0% (*p* = 0.56) in concentric stenosis confirms the significant effect of IVL in calcified coronary lesions.

**Conclusion:**

In this first report from a pooled patient-level analysis of coronary IVL from the Disrupt CAD I and CAD II studies, IVL use was associated with consistent improvement in procedural and clinical outcomes in both eccentric and concentric calcified lesions.

## Introduction

Severe calcification of coronary stenoses still provides a major challenge for percutaneous coronary intervention (PCI). To avoid a sub-optimal clinical outcome, it is important to achieve sufficient luminal gain during lesion preparation prior to stent implantation [1, 2]. Besides the risk of impaired stent expansion, severe coronary calcification may also lead to sub-optimal PCI outcomes by limiting lesion crossing, altering drug elution kinetics, and interfering with optimal stent expansion [3–7].

Intravascular lithotripsy (IVL) has been recently introduced to modify calcified coronary plaques and is useful in overcoming some of the limitations of the more commonly used techniques, e.g., percutaneous transluminal coronary angioplasty (PTCA) with non-compliant (NC) balloons, cutting-/scoring-balloons, and rotational atherectomy (RA). NC balloon dilatation, even with high pressure, is often insufficient to apply the necessary force for disrupting calcifications. Due to the eccentricity of calcified lesions, balloon dilatation often results in disruption or dissection of healthy intima or fibrous plaques rather than modification of calcified segments within the artery [8]. Cutting and scoring balloons, though able to debulk the lesion more intensely than NC balloons suffer from the same limitation. Even rotational or orbital atherectomy (OA), the most effective techniques for modification of calcified plaques available prior to IVL, are limited due to guidewire bias, which may result in inhomogeneous ablation leaving significant areas of the calcified plaques unmodified, particularly in eccentric lesions [9]. Additionally, periprocedural complications including slow-/no-flow, coronary perforation, periprocedural myocardial infarction occur more frequently with atherectomy techniques as compared to balloon techniques [10, 11].

IVL catheters are equipped with emitters that deliver pulsatile sonic pressure waves circumferentially to the vessel wall. IVL catheters are equipped with emitters along with the balloon that delivers pulsatile shockwaves to the surrounding plaque after activation. An electrical discharge vaporizes the fluid within the balloon to generate a rapidly expanding bubble and collapses within a few microseconds afterward. Soft tissue transmits the pulsatile mechanical energy, while microfractures are induced in rigid calcified structures and thus break up the calcified plaques. The treatment sequence takes 10 s, during which shockwaves are emitted at a frequency of 80 Hz. 2–4 sequences are performed per vessel section. This provides the unique opportunity to modify the calcified plaque homogenously and reach calcification even in deeper vessel layers. The aim of this study was to assess the safety and effectiveness of IVL in treating eccentric calcified coronary lesions.

## Methods

### Study design and population

Between December 2015 and March 2019, 180 patients were enrolled in the Disrupt CAD I and Disrupt CAD II studies across 19 sites in 10 countries. Disrupt CAD I (*n* = 60) was a pre-market, prospective, single-arm, multi-center study designed to evaluate the safety and performance of the Shockwave (Shockwave Medical Inc., Santa Clara, CA, USA) coronary intravascular lithotripsy (IVL) system in the treatment of calcified coronary lesions for the purpose of optimizing the placement of stents and reducing the ultimate residual stenosis [12].

Disrupt CAD II (*n* = 120) was a post-market study evaluating the safety and performance of the coronary IVL system following expansion to a broader patient population and additional physician users [13]. The inclusion and exclusion criteria for both studies were identical and included patients with significant native calcified coronary artery disease suitable for PCI. In both studies, patients were required to have a single target lesion requiring PCI with diameter stenosis ≥ 50%, lesion length ≤ 32 mm in native coronary arteries, and severe calcification as determined by the operators, defined as calcification within the lesion on both sides of the vessel assessed by angiography. Primary endpoints of the Disrupt CAD I study were freedom from major adverse cardiac events (MACE) within 30 days of the procedure. MACE was defined as cardiac death, myocardial infarction or target vessel revascularization (TVR). IVL performance was defined as the ability of the IVL system to produce residual stenosis of < 50% after stenting without intra-hospital MACE [12]. The primary endpoint of the Disrupt CAD II study was the frequency of in-hospital MACE [13].

The same independent angiographic core lab was utilized for both studies and analyzed all procedural angiograms (Yale Cardiovascular Research Group, New Haven, CT, USA). The angiographic core lab defined an eccentric lesion as a stenotic lesion that had one of its luminal edges in the outer one-quarter of the apparent normal vessel lumen [14–16]. Concentric lesions were defined using the same criteria while involving both luminal edges. Whenever possible, multiple angiographic angles were used to confirm the lesion classification.

All patients gave written informed consent before enrollment. The study was conducted in accordance with the Declaration of Helsinki and applicable laws by all related governmental bodies. Studies were registered at https://www.clinicaltrials.gov; their unique identifiers: NCT02650128 and NCT03328949.

### Study device

The coronary IVL system is a 6Fr compatible semi-compliant balloon catheter, containing two electrically charged lithotripsy emitters, inserted over a rapid exchange 0.014″ guidewire [11–13]. Balloon catheters are available in several diameters (2.5–4.0 mm in steps of 0.5 mm) with a length of 12 mm. The balloon is expanded to 4 atm by a fluid (50:50 mixture of NaCl 0.9% and contrast media) optimized to transmit circumferential sonic pressure waves through soft vascular tissue. A small electrical discharge at the emitters vaporizes this fluid, thereby generating a rapidly expanding and collapsing bubble within the balloon. The resulting mechanical energy (approximately 50 atm) selectively induces fractures in the calcium. The IVL system allows the manual application of individual therapy cycles, each comprising 10 pulses (one pulse per second) in series, with a maximum of eight cycles emitted by each catheter [13, 17, 18].

### Study procedure

PCI was performed via 6Fr or larger femoral or radial access. The IVL catheter was inserted using a standard 0.014″ guidewire. If passing the IVL device was initially unable to cross the target lesion, preparation with a small NC balloon (1.5 mm diameter), buddy wire-technique or guidewire extension was allowed per protocol. Lithotripsy-balloon diameter was selected 1:1 according to the angiographically estimated reference lumen diameter. After positioning, the balloon was inflated to 4 atm to achieve proper contact with the vessel wall and one cycle was delivered; the balloon was inflated to 6 atm subsequently. Treatment cycles were repeated as necessary to cover the whole lesion. If the maximum of eight cycles (80 pulses) had been delivered without sufficient lesion preparation, the use of additional IVL catheters with the same or larger diameters was allowed per protocol. Stent implantation and post-dilatation were performed according to the standard of care in each institution. Post-procedure medication and selection of dual antiplatelet therapy were at the discretion of the operator. Clinical follow-up was conducted 30 days post-procedure by standardized telephone interview.

### Statistical analysis

Patient baseline characteristics and procedural data were analyzed and represented using frequency, mean, SD, and median. In comparing two groups, the *t* test or Wilcoxon sum test was utilized for continuous variables and Fisher’s exact test for dichotomous variables. All statistical tests were two-sided, with *p* values < 0.05 considered statistically significant. Statistical analyses were performed using SAS (SAS Institute, Cary, NC, USA), version 9.4.

## Results

### Patient data

Patient-level data were pooled from Disrupt CAD I and Disrupt CAD II study with eccentric lesions identified in 47 patients and concentric lesions in 133 patients. Mean patient age was 72.1 ± 9.7 years. There were no significant differences between patients with eccentric or concentric lesions regarding baseline characteristics (Table 1). There was a trend towards higher frequencies of previous myocardial infarction (40.4% vs. 27.1%; *p* = 0.10), arrhythmias (31.9% vs. 18%; *p* = 0.06) and renal insufficiency (14.9% vs. 6.8%; *p* = 0.13) in patients with eccentric stenoses as compared to patients with concentric stenoses.

**Table 1:** Baseline characteristics

	Overall (*n* = 180)	Eccentric (*n* = 47)	Concentric (*n* = 133)	*p* value
Age	72.1 ± 9.7	73.0 ± 10.1	71.8 ± 9.6	0.35
Male	142 (78.9)	38 (80.9)	104 (78.2)	0.84
Diabetes	56 (31.1)	17 (36.2)	39 (29.3)	0.49
Hypertension	144 (80.0)	39 (83.0)	105 (78.9)	0.70
Hyperlipidemia	134 (74.4)	34 (72.3)	100 (75.2)	0.85
Renal Insufficiency	16 (8.9)	7 (14.9)	9 (6.8)	0.13
MI^a^	55 (30.6)	19 (40.4)	36 (27.1)	0.10
Arrhythmia	39 (21.7)	15 (31.9)	24 (18.0)	0.06

Values are mean ± standard deviation or *n* (%)

^a^*MI* myocardial infarction

### Lesion characteristics

Target lesions were located in the left anterior descending artery in 57.2%, in the right coronary artery in 29.5%, in the circumflex artery in 12.2%, and in the protected left main in 1.1%. There were no significant differences between groups regarding the lesion location. However, patients with eccentric lesions demonstrated significantly larger reference vessel diameter (RVD, 3.2 ± 0.6 mm vs. 3.0 ± 0.5 mm; *p* = 0.04 and significantly shorter lesion length (16.7 ± 7.0 mm vs. 20.9 ± 10.7 mm; *p* = 0.01) as compared to patients with concentric lesions. For detailed lesion characteristics see also Table 2.

**Table 2:** Lesion characteristics

	Overall (*n* = 180)	Eccentric (*n* = 47)	Concentric (*n* = 133)	*p* value
Target vessel				
Protected LM^a^	2 (1.1)	2 (4.3)	0 (0.0)	0.10
LAD^b^	103 (57.2)	23 (48.9)	80 (60.2)	
Cx^c^	22 (12.2)	3 (6.4)	19 (14.3)	
RCA^d^	53 (29.4)	19 (40.4)	34 (25.6)	
RVD^e^ (mm)	3.0 ± 0.5	3.2 ± 0.6	3.0 ± 0.5	0.03
MLD^f^ (mm)	1.1 ± 0.4	1.2 ± 0.5	1.1 ± 0.4	0.11
DS^g^ (%)	62.7 ± 12.9	61.7 ± 14.1	63.1 ± 12.5	0.44
Lesion length (mm)	19.8 ± 10.0	16.7 ± 7.0	20.9 ± 10.7	0.04
Calcified length (mm)	24.6 ± 12.5	24.2 ± 15.7	24.8 ± 11.3	0.18
Severe calcification	161 (89.4)	41 (87.2)	120 (90.2)	0.77

Values are mean ± standard deviation or *n* (%). Severe calcification was confirmed by angiography when radiopacity was noted without cardiac motion prior to contrast injection

^a^*LM* left main

^b^*LAD* left anterior descending artery

^c^*Cx* circumflex artery

^d^*RCA* right coronary artery

^e^*RVD* reference vessel diameter

^f^*MLD* minimum lumen diameter

^g^*DS* diameter stenosis

### Procedural characteristics

Pre-dilatation was performed in 40% of patients and post-dilatation in 81.7%. Mean procedure time was 76.5 ± 37.0 min, mean fluoroscopy time 22.7 ± 15.9 min, and mean contrast load 207.3 ± 87.5 ml. The number of IVL treatment cycles delivered was heavily left-skewed, with a median of 68.5 [40, 80] IVL pulses delivered, translating to 3.5 [2.5, 5.8] IVL pulses/mm of lesion length; this was consistent between patients with eccentric and concentric lesions. Intravascular imaging using optical coherence tomography was performed in 78 patients (43%). No procedural characteristics differed significantly between groups. See also Table 3.

**Table 3:** Procedural characteristics

	Overall (*n* = 180)	Eccentric (*n* = 47)	Concentric (*n* = 133)	*p* value
Procedure time (min)	76.5 ± 37.0	74.6 ± 40.1	77.2 ± 36.0	0.46
Fluoroscopy time (min)	22.7 ± 15.9	19.8 ± 13.5	23.8 ± 16.6	0.35
Contrast volume (ml)	207.3 ± 87.5	191.5 ± 87.7	212.8 ± 87.0	0.07
IVL^a^ catheters (*n*)	1.5 ± 0.9	1.3 ± 0.6	1.5 ± 0.9	0.22
IVL^a^ pulses (*n*)	68.5 [40, 80]	60 [50, 80]	70 [40, 80]	0.79
Pulses/mm lesion length	3.5 [2.5, 5.8]	3.7 [2.9, 6.5]	3.4 [2.3, 5.1]	0.13
Max IVL^a^ inflation pressure	5.8 ± 0.8	6.0 ± 0.7	5.8 ± 0.8	0.15
Number of stents (*n*)	1.4 ± 0.7	1.3 ± 0.5	1.4 ± 0.7	0.12
Pre-dilatation	72 (40.0)	20 (42.6)	52 (39.1)	0.81
Post-dilatation	147 (81.7)	36 (76.6)	111 (83.5)	0.41

Values are mean ± standard deviation or median [Q1, Q3] or *n* (%)

^a^*IVL* intravascular lithotripsy

### Outcome

**Fig. 1:**
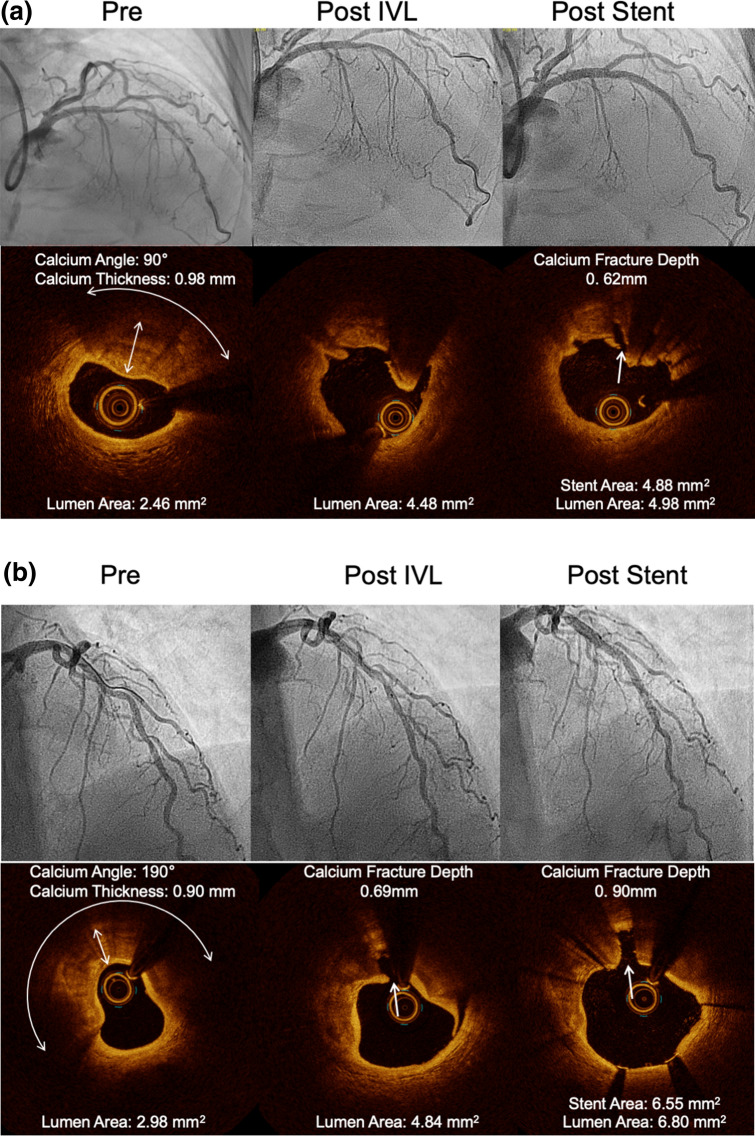
IVL in eccentric and concentric coronary lesions. Representative angiography and optical coherence tomography images from cases involving an **a** eccentric lesion and **b** concentric lesion. Fractured calcium is visible within the intimal and medial vessel layers for both lesions. In each example, increased lumen area is notable post IVL treatment and again post stent

Clinical success, defined as final post-stent residual stenosis < 50% after stenting with no in-hospital MACE, was achieved in 93.3% of patients (Eccentric: 93.6% vs. concentric 93.2%; *p* = 0.80). Angiographic success, defined as success in facilitating stent delivery with < 50% residual stenosis and without major angiographic complications (severe dissection impairing flow [type D–F], perforation, abrupt closure, persistent slow flow, or no reflow), was achieved in 98.9% of patients (Eccentric: 100% vs. concentric 98.5%; *p* = 0.97). An exploratory goal of < 30% residual stenosis was achieved with high frequency in both groups (Eccentric: 97.9% vs. concentric 97.0%; *p* = 0.84). Residual percent diameter stenosis (Eccentric: 61.7 ± 14.1% vs. concentric: 63.1 ± 12.5%; *p* = 0.44) and acute gain (Eccentric: 1.8 ± 0.5 mm vs. concentric: 1.7 ± 0.5 mm; *p* = 0.47) were similar between groups. Representative angiographic and optical coherence tomography images from eccentric and concentric lesions are shown in Fig. 1.

After IVL, no perforations, abrupt closure, slow-flow or no-reflow events were observed in either group, and low rate of flow-limiting dissections (Grade D–F: Eccentric 0% vs. concentric 1.7%, *p* = 0.54) occurred. All dissections were resolved with stent delivery.

During in-hospital follow-up, there were two cases (4.3%) of non-Q-wave myocardial infarction in patients with eccentric lesions and eight cases (6.0%) in patients with concentric lesions (*p* = 0.93). Neither group experienced cardiac death or target vessel revascularization during in-hospital follow-up.

The 30-day MACE rate was 8.7% in patients with eccentric lesions and 6.0% in patients with concentric lesions (*p* = 0.80). No new observations of non-Q-wave myocardial infarction were observed following hospital discharge. There was one cardiac death in the group with eccentric lesions. The cardiac death occurred in a 70-year-old who originally presented with pre-syncope and died suddenly 14 days after treatment of a 95% lesion in the distal right coronary artery. The inclusion of this patient was a protocol deviation, as the patient met defined angiographic exclusion criterion (second lesion with ≥ 50% stenosis in the same target vessel) due to occluded posterior descending coronary artery and reference vessel diameter > 4.0 mm (quantitative coronary angiography: 4.57 mm) [15]. There were no significant differences in the frequency of 30-day MACE when comparing patients with eccentric and concentric lesions. For detailed outcome data see also Tables 4, 5, 6 and 7.

**Table 4:** Performance outcomes

	Overall (*n* = 180)	Eccentric (*n* = 47)	Concentric (*n* = 133)	*p* value
Clinical success	168 (93.3)	44 (93.6)	124 (93.2)	1.0
Angiographic success	178 (98.9)	47 (100.0)	131 (98.5)	1.0
Stent delivery	180 (100.0)	47 (100.0)	133 (100.0)	
Final in-stent angiographic outcomes				
MSD^a^ (mm)	2.8 ± 0.5	3.0 ± 0.5	2.7 ± 0.5	0.004
Residual stenosis (%)	9.7 ± 9.2	8.6 ± 9.8	10.0 ± 9.0	0.56
Acute gain (mm)	1.7 ± 0.5	1.8 ± 0.5	1.7 ± 0.5	0.47
Residual stenosis < 50%	180 (100.0)	47 (100.0)	133 (100.0)	
Residual stenosis < 30%	175 (97.2)	46 (97.9)	129 (97.0)	0.84

Values are mean ± standard deviation or *n* (%)

^a^*MSD* minimum stent diameter

**Table 5:** Angiographic complications—Post-IVL

	Overall (*n* = 161)	Eccentric (*n* = 44)	Concentric (*n* = 117)	*p* value
Dissections, type D–F	2 (1.2)	0 (0.0)	2 (1.7)	0.54
Perforation	0 (0.0)	0 (0.0)	0 (0.0)	
Abrupt closure	0 (0.0)	0 (0.0)	0 (0.0)	
Slow flow	0 (0.0)	0 (0.0)	0 (0.0)	
No reflow	0 (0.0)	0 (0.0)	0 (0.0)	

Values are *n* (%)

**Table 6:** Angiographic complications—Final

	Overall (*n* = 180)	Eccentric (*n* = 47)	Concentric (*n* = 133)	*p* value
Dissections, type				
D–F	0 (0.0)	0 (0.0)	0 (0.0)	
Perforation	0 (0.0)	0 (0.0)	0 (0.0)	
Abrupt closure	0 (0.0)	0 (0.0)	0 (0.0)	
Slow flow	0 (0.0)	0 (0.0)	0 (0.0)	
No reflow	0 (0.0)	0 (0.0)	0 (0.0)	

Values are *n* (%)

**Table 7:** MACE

	Overall (*n* = 180)	Eccentric (*n* = 47)	Concentric (*n* = 133)	*p* value
In-hospital	10 (5.6)	2 (4.3)	8 (6.0)	0.93
Cardiac death	0 (0.0)	0 (0.0)	0 (0.0)	
Non-Q-wave MI^a^	10 (5.6)	2 (4.3)	8 (6.0)	
Q-wave MI^a^	0 (0.0)	0 (0.0)	0 (0.0)	
TVR^b^	0 (0.0)	0 (0.0)	0 (0.0)	
30-day*	12 (6.7)	4 (8.7)	8 (6.0)	0.80
Cardiac death	1 (0.6)	1 (2.2)	0 (0.0)	
Non-Q-wave MI^a^	10 (5.6)	2 (4.3)	8 (6.0)	
Q-wave MI^a^	1 (0.6)	1 (2.2)	0 (0.0)	
TVR^b^	1 (0.6)	1 (2.2)	0 (0.0)	

Values are *n* (%)

^a^*MI* myocardial infarction

^b^*TVR* target vessel revascularization

*One subject with two events; one subject withdrew prior to the 30-day end-point

## Discussion

The main findings of this pooled patient-level analysis are that IVL treatment of eccentric coronary lesions is associated with consistent outcomes including high procedure success and low vascular complications and that there are no significant differences regarding procedural and clinical outcome when comparing IVL treatment of eccentric with concentric lesions.

IVL provides a unique therapy to modify calcified coronary plaques even in deeper vessel layers [12, 13]. IVL mechanical pressure waves are transduced through the soft tissue of the vessel wall. Rigid calcifications cannot transduce this mechanical energy, so the energy selectively fractures calcified plaque. All other debulking techniques (Cutting-/Scoring-Balloon, atherectomy techniques) may suffer from guidewire bias leading to inhomogeneous plaque modification [9, 19–21]. OA and RA modify calcified plaque by generating a relatively smooth, circular channel, strictly following the guidewire [19, 21–24]. This facilitates balloon or stent delivery, although the gain in the cross-sectional area is modest [24]. Nevertheless, it must be kept in mind, that this atherectomy “tunnel” may not be located centrally in the coronary artery which can result in asymmetric stent expansion and undesirable clinical outcomes. Furthermore, in regions with tortuosity and eccentric lesions, there is a significant risk of coronary perforation with OA or RA [25] and damage to healthy portions of the vessel wall. Eccentric coronary calcifications are particularly difficult to modify by OA or RA atherectomy due to the guidewire being displaced away from the target lesion. In comparison, the IVL balloon inflated to 4 atm is in contact with all parts of the surrounding vessel and therefore has no guidewire bias. This provides the possibility for homogeneous plaque modification, in both the intima and the media, especially in eccentric coronary plaques that are usually associated with suboptimal outcomes after PCI [26, 27]. Patient age has continuously increased over the past years resulting in higher calcium burden of coronary plaques as well as more complex coronary stenoses, such as eccentric calcified coronary lesions. The increasing frequency of these complex lesions is associated with an impaired clinical outcome [28]. Therefore, promising treatment options for eccentric calcified lesions, such as IVL, need to be evaluated and established in clinical routine. IVL has demonstrated effective treatment of calcifications located in deeper vessel layers [13, 29]. Circumferential plaque modification results in increased vessel compliance, demonstrated by increasing vessel diameter during constant balloon pressure [18]. As a result, IVL facilitates full, symmetrical stent expansion [13].

As the utilization of IVL has increased, there has been much discussion as to which clinical cases the technology is best suited for. In this first comparison between eccentric and concentric lesions, as defined angiographically, it is instructive to observe high procedural success rates with IVL regardless of lesion type. Angiographic success was achieved in 100% of eccentric lesions and 98.5% of concentric lesions, which emphasizes the effectiveness of IVL in treating a wide range of stenoses. This clinical success was achieved using a similar number of pulses/mm lesions among the two groups in this study. Per protocol, the goal was to deliver lithotripsy until < 50% residual stenosis was achieved, but ending therapy was at physician’s discretion and there were no limitations to the absolute number of pulses delivered. Regardless of the variability in the strategy of pulse delivery, the number of pulses delivered, and the pulses/mm lesion was similar between eccentric and concentric lesions. Accordingly, this analysis demonstrates no limitations in the efficiency or effectiveness of IVL treatment in eccentric lesions. Based on the results of this study, IVL should be considered as a valuable treatment option in both eccentric and concentric calcified lesions. This seems to be even more important when considering that rotational or orbital atherectomy, the most effective techniques prior to IVL, is limited due to guidewire bias, which may result in inhomogeneous ablation leaving significantly unmodified areas in eccentric lesions. Moreover, it may be suited to make safe and effective plaque modulation in calcified coronary stenoses available for a wider patient collective than it is at the moment since atherectomy techniques are only used in a small part of patients in need due to their technical complexity [30, 31].

According to our results, Mattesini et al. reported similar results for IVL treatment of eccentric (Calcium arc > 180°) and concentric calcified stenosis (Calcium arc ≤ 180°) when comparing the acute results using intravascular imaging by optical coherence tomography in a prospective registry including 28 patients [32]. There were no significant differences regarding in-stent minimum lumen diameter, in-stent minimal lumen area, and the acute gain when comparing eccentric and concentric calcified stenoses. If this really translates into lower adverse event rates and better clinical outcome needs to be evaluated in future clinical trials.

### Limitations

Our study has a number of limitations. First, this is a retrospective pooled analysis from two different studies that were designed for evaluating the safety and procedural success of IVL. Nevertheless, the inclusion and exclusion criteria of both studies were identical and data analysis was performed by the same independent core lab. Patient numbers were quite small with 180 patients being included in the aggregated studies. Nonetheless, this study includes the largest number of patients with coronary IVL treatment performed so far.

A further limitation is the angiographic endpoint of clinical success, defined as residual diameter stenosis of less than 50% after IVL and stenting, which is quite conservative. The endpoint was chosen as equivalent to the ORBIT II study which was used as a primary comparator for the Disrupt CAD I study [33]. Nonetheless, the final residual stenosis of 8.6 ± 9.8% in eccentric and 10.0 ± 9.0% (*p* = 0.56) in concentric stenosis confirms the significant effect of IVL on these lesions. Additionally, given the angiographic limitations in determining the exact arc of calcium within the lesions, further insights from planned OCT and IVUS analyses from the Disrupt CAD clinical program will add additional valuable insights.

Future clinical trials should focus on comparing IVL and other debulking techniques for the treatment of calcified coronary lesions to evaluate the technique in comparison to the current standards of care. No according data have been published so far.

## Conclusion

In this first report from a pooled patient-level analysis of coronary IVL from the Disrupt CAD I and CAD II studies, IVL use was associated with high procedural success and consistent clinical outcomes in both eccentric and concentric calcified lesions.
